# Uremic Vascular Calcification Is Correlated With Oxidative Elastic Lamina Injury, Contractile Smooth Muscle Cell Loss, Osteogenesis, and Apoptosis: The Human Pathobiological Evidence

**DOI:** 10.3389/fmed.2020.00078

**Published:** 2020-03-24

**Authors:** Jia-Feng Chang, Shih-Hao Liu, Kuo-Cheng Lu, Shuk-Man Ka, Chih-Yu Hsieh, Chun-Ta Ho, Wei-Ning Lin, Li-Li Wen, Jian-Chiun Liou, Shu-Wei Chang, Chang-Chin Wu, Ting-Ming Wang, Yen-Yao Li

**Affiliations:** ^1^Division of Nephrology, Department of Internal Medicine, Taipei Medical University-Shuang Ho Hospital, New Taipei City, Taiwan; ^2^Department of Pathology, Tri-Service General Hospital, National Defense Medical Center, Taipei, Taiwan; ^3^Graduate Institution of Biomedical and Pharmaceutical Science, College of Medicine, Fu Jen Catholic University, New Taipei City, Taiwan; ^4^Department of Nursing, Yuanpei University of Medical Technology, Hsinchu, Taiwan; ^5^Renal Care Joint Foundation, New Taipei City, Taiwan; ^6^Division of Nephrology, Department of Internal Medicine, En Chu Kong Hospital, New Taipei City, Taiwan; ^7^Academy of Medicine, National Defense Medical Center, Graduate Institute of Aerospace and Undersea Medicine, Taipei, Taiwan; ^8^Division of Pathology, En-Chu-Kong Hospital, New Taipei City, Taiwan; ^9^College of Medicine, Graduate Institute of Clinical Medicine, Taipei Medical University, Taipei, Taiwan; ^10^Division of Nephrology, Department of Medicine, Fu Jen Catholic University Hospital, School of Medicine, Fu Jen Catholic University, New Taipei City, Taiwan; ^11^Department of Medical Laboratory Science and Biotechnology, Yuanpei University, Hsinchu, Taiwan; ^12^Department of Clinical Laboratory, En Chu Kong Hospital, New Taipei City, Taiwan; ^13^School of Biomedical Engineering, Taipei Medical University, Taipei, Taiwan; ^14^Department of Civil Engineering, National Taiwan University, Taipei, Taiwan; ^15^Department of Biomedical Engineering, Yuanpei University of Medical Technology, Hsinchu, Taiwan; ^16^Department of Orthopaedic Surgery, En-Chu-Kong Hospital, New Taipei City, Taiwan; ^17^Department of Orthopaedic Surgery, School of Medicine, National Taiwan University, Taipei, Taiwan; ^18^Department of Orthopaedic Surgery, National Taiwan University Hospital, Taipei, Taiwan; ^19^Department of Orthopedic Surgery, Chang Gung Memorial Hospital, Chiayi City, Taiwan; ^20^College of Medicine, Chang Gung University, Taoyuan, Taiwan

**Keywords:** uremic vascular calcification, oxidative injury, elastic lamina, contractile smooth muscle cell, osteogenesis, apoptosis

## Abstract

**Background:** Uremic vascular calcification (UVC) is reminiscent of osteogenesis and apoptosis in vascular smooth muscle cell (VSMC). We aimed to identify how circulating procalcific particles dramatically leak into VSMC layer in human tissue models of vascular rings.

**Methods:** According to baseline estimated glomerular filtration rate (eGFR), patients following lower extremity amputation were divided into three groups: normal renal function (eGFR ≧ 60 ml/min), mild-to-moderate (15 ml/min < eGFR ≧ 60 ml/min) and severe chronic kidney disease (CKD) (eGFR ≦ 15 ml/min). Arterial specimens with immunohistochemistry stain were quantitatively analyzed for UVC, internal elastic lamina (EL) disruption, α-SMA, osteogenesis, apoptosis, and oxidative injury. Correlations among UVC severity, eGFR, EL disruption, osteogenesis, and oxidative injury were investigated.

**Results:** CKD arteries were associated with eGFR-dependent EL disruption corresponding to UVC severity. CKD arteries exhibited lower α-SMA, higher expressions of caspase-3 and terminal deoxynucleotidyl transferase dUTP nick end labeling (TUNEL), indicative of contractile VSMC loss, and apoptosis. Enhanced expressions of alkaline phosphatase and Runx2 were presented in VSMCs of CKD arteries, indicative of osteogenic differentiation. Above eGFR-dependent UVC and EL disruption correlated expressions of 8-hydroxy-2′-deoxyguanosine (8-OHdG), indicating oxidative EL injury promoted procalcific processes.

**Conclusions:** Circulating uremic milieu triggers vascular oxidative stress, leading to progressive internal EL disruption as a key event in disabling VSMC defense mechanisms and catastrophic mineral ion influx into VSMC layer. Oxidative EL injury begins in early CKD, corresponding with active VSMC re-programming, apoptosis, and ultimately irremediable UVC. In light of this, therapeutic strategies targeting oxidative tissue injury might be of vital importance to hinder the progression of UVC related cardiovascular events.

## Introduction

Cardiovascular diseases (CVD) still top the list as the leading causes of death in patients with chronic kidney disease (CKD) worldwide (1, 2). CKD related uremic milieu accelerates the decline rate of CVD and premature cardiovascular (CV) death, yet uremic vascular calcification (UVC) serves as a pivotal contributor (2, 3), beginning in the first decade of life in children on dialysis (4). Medial UVC induces cardiovascular (CV) stiffening, resulting in systolic hypertension, reduced coronary perfusion, and heart failure with preserved ejection fraction (5). Dialysis patients with UVC often do not respond effectively to standard CV medications, such as statins and antihypertensive treatments (6, 7). Accordingly, there is a serious unmet need to develop therapies that target the pathomechanism of UVC in clinical practice.

The severity of arterial stiffness and UVC is in parallel with all-cause and particular CV death in patients with end-stage renal disease (ESRD) (8). The reasons why ESRD patients are at particular risk for UVC are intricate, including chronic mineral dysregulation (9), calcium-based therapies, pro-oxidant and pro-inflammatory effects of uremic toxins, active process of osteogenesis in vascular smooth muscle cells (VSMC) and passive process of mineral deposition in extracellular matrix (ECM) (10). Emerging evidence have highlighted that UVC is a cell-mediated active process associated with VSMC death, maladaptation, and phenotypic modulation to promote UVC progression (11–13). Moreover, vascular oxidative injury is attributed to an imbalance between the excess reactive oxygen species (ROS) generation and inadequate anti-oxidant defense forces (14). Oxidative stress derived from uremic toxins initiates tissue injury by inducing damages in both proteins and DNA, suppressing VSMC repair functions, and a vicious circle in activation of mitochondrial ROS (15–17). If above documented mechanisms are true, how do circulating mineral ions dramatically leak into the arterial medial layer? The last piece of the puzzle and key players in CKD-driven UVC are still lacking, leading to a therapeutic dilemma. Given comprehensive pictures of UVC remain elusive in cell models of *in vitro* research, we aimed to investigate the association between UVC and oxidative tissue injury using human models of vascular rings in this study.

## Methods

### Patients and Amputation Specimens

The study had been approved by the Research Ethics Review Committee of the En Chu Kong Hospital (ECKH1050402) in accordance with the ethical standards of the committee and the Helsinki declaration for research in humans. Based on the clinical history obtained from the electronic medical records between January 2012 and December 2016, patients who underwent lower limb amputation surgery were enrolled in this study. The underlying diseases included acute limb ischemia, critical limb ischemia, gangrene, traumatic limb amputation, and so on. The amputation specimens of lower-extremity arteries were included for further investigation. The following bio-clinical and laboratory parameters of study patients were recorded at baseline: age, gender, systolic blood pressure, diastolic blood pressure, blood urea nitrogen (BUN), creatinine (Cr), estimated glomerular filtration rate (eGFR), glucose, albumin, sodium, potassium, calcium, phosphate, adjusted calcium [calcium + (4.0—albumin in g/dL) × 0.8], calcium-phosphate product (adjusted calcium × phosphate), alkaline phosphatase (ALP), alanine aminotransferase, aspartate aminotransferase, total cholesterol, triglyceride, low density lipoprotein, and C-reactive protein (CRP). Patients with incomplete data were excluded from the study. Pathological sections with medium-sized muscular arteries from patients underwent amputation surgery were available for analysis. All patients were then stratified by CKD staging into the following three groups for further investigation: severe CKD (eGFR ≦ 15 ml/min), mild-to-moderate CKD (15 ml/min < eGFR ≦ 60 ml/min); and controls (eGFR > 60 ml/min). Circulating procalcific particles were defined as calcium, phosphate, and precipitation of calcium phosphate.

### Immunohistochemistry (IHC) Staining and Digital Imaging

**Figure 1:**
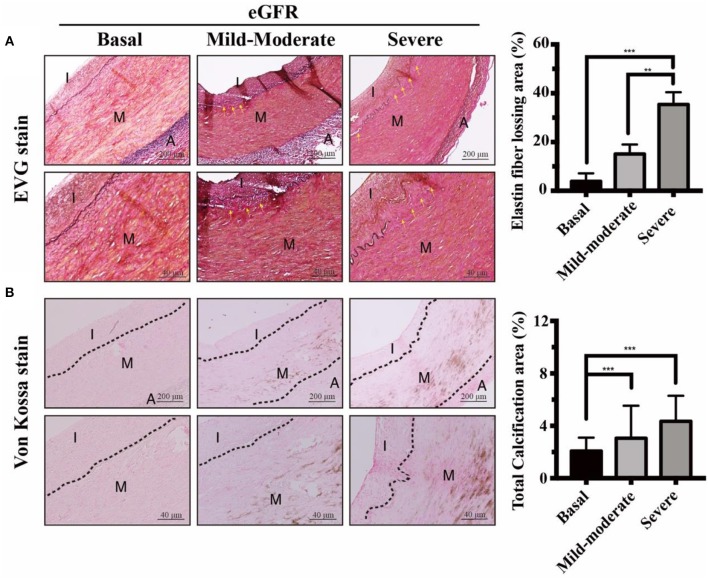
Effects of eGFR-dependent internal EL loss on UVC in CKD arteries. Muscular arteries were classified into three study groups of normal controls, mild-to-moderate and severe CKD. Control groups, eGFR ≧ 60 ml/min; Mild-to-moderate CKD, 15 ml/min < eGFR ≦ 60 ml/min; Severe CKD, eGFR ≦ 15 ml/min. **(A)** Internal EL defect areas were examined using EVG stain. Yellow arrows indicated internal EL loss in mild-to-moderate and severe CKD groups, compared to normal controls. Scale bars in the upper panels are 200 μm and 40 μm in the lower panels. **(B)** Calcium deposits were localized using Von Kossa stain. Note that stretching, fragmentation and disruption of internal EL were presented adjacent to the eGFR-dependent UVC regions. Fifty-one human samples were used for the experiments: controls (*n* = 19); mild-to-moderate CKD (*n* = 15); severe CKD (*n* = 17). For image analyses, three random high-resolution fields were obtained in each sample. Quantitative analyses of EVG and Von Kossa staining were performed using ImageJ software. Data are expressed as mean ± SD. **P < 0.01; ***P < 0.001. A, adventitia; CKD, chronic kidney disease; eGFR, estimated glomerular filtration rate; EL, elastic lamina; EVG, elastic tissue fibers-Verhoeff's Van Gieson; I, intima; M, media; UVC, uremic vascular calcification.

Histopathological characterizations of all amputation specimens were evaluated in detail. The tissue morphology was assessed by hematoxylin and eosin stain. Briefly, tissue sections measuring 3 μm were created from the paraffinize blocks, de-paraffinized in xylene and rehydrated in a graded alcohol series. The images were captured under Nikon Digital Camera Microscope (Nikon, Tokyo, Japan). Our staining procedures were conducted in accordance with the Kit User Guide completely. The histological sections of human artery vessel rings were stained with (1) Von Kossa (CVK-1-IFU, ScyTek Laboratories, UT, USA.) for calcification; (2) elastic tissue fibers—Verhoeff's Van Gieson (EVG) stain (Ab150667, Abcam, Cambridge, UK. 1:200 dilution) for elastic tissue fibers; (3) alpha-smooth muscle actin (αSMA) (Sc-53142, Santa Cruz, CA, USA. 1:200 dilution) for VSMC contractility; (4) immunohistochemistry performed for 8-hydroxy-2'-deoxyguanosine (8-OHdG) (Bs-1278R, Bioss Antibodies, MA, USA. 1:200 dilution) for DNA damage, (5) alkaline phosphatase (Ab108337, Abcam, Cambridge, UK. 1:200 dilution); (6) RUNX2 (Sc-101145, Santa Cruz, CA, USA. 1:200 dilution) for osteogenic differentiation. Apoptosis was examined by immunohistochemistry using a cleaved caspase-3 antibody (#9961, Cell Signaling Technology, MA, USA. 1:200 dilution) and terminal deoxynucleotidyl transferase dUTP nick end labeling (TUNEL) (#17-141, EMD Millipore, CA, USA.). The procedure of performing immunohistochemical staining was designed according to the manufacturer's protocol (BioTnA, Kaohsiung, Taiwan). The blocking step before primary antibodies is necessary (TA00C2, BioTnA, Kaohsiung, Taiwan). Tissue sections stained with primary antibodies, followed by HRP-conjugated anti-rabbit secondary antibody (TAHC02D, BioTnA, Kaohsiung, Taiwan). The expression levels were detected by using the TAlink mouse/rabbit polymer detection system (TAHC04D, BioTnA, Kaohsiung, Taiwan). All glass slides of tissue sections were converted to digital virtual slides via the slide scanner (Motic Easyscan Digital Slide Scanner, Hong Kong, China) with high precision autofocus. A 20X/NA 0.65 objective was used with a 0.5 μm/pixel resolution. The IHC staining results were reviewed by a blinded pathologist (Dr. Shih-Hao Liu) at the En Chu Kong Hospital (New Taipei City, Taiwan). Regions of interest were marked and expressed as percentages of total areas around the Von Kossa positive areas within the vessels. The procedure of performing Von Kossa and EVG staining was designed according to the manufacturer's instructions. For Von Kossa staining, we deparaffinized sections and hydrate to distilled water. Slides were then incubated in Silver Nitrate Solution (5%) for 30–60 min while exposing to either ultraviolet light or incandescent light at 75 watts or greater. For best results, we kept light source within 2 feet (61 cm) of slide during Silver Nitrate staining procedure. Afterwards, slides were rinsed in three changes of distilled water and incubated in Sodium Thiosulfate Solution (5%) for 2 min. After the step of rinse for 2 min in running tap water followed by two changes of distilled water, the tissue section was stained with Nuclear Fast Red Solution for 5 min. Next, we rinsed slides for 2 min in running tap water followed by 2 changes of distilled water. Finally, slides were mounted with synthetic resin after quick dehydration in the absolute alcohol. The calcification area was quantified and expressed as a percentage of Von Kossa positive area (brown color) divided by the area of arterial medial layer (M) (Figure 1). For EVG staining, slides were placed in working Elastic Stain Solution for 15 min after deparaffinization. Slides were then rinsed in running tap water until no excess stain remains on slide. After the step of dip in differentiating solution 15–20 times and rinse in tap water, we checked slides microscopically for proper differentiation and rinsed slides in running tap water. Next, slides were mounted in Sodium Thiosulfate Solution for 1 min and rinsed in running tap water. Afterwards, we stained slides using Van Gieson's Solution for 2–5 min and rinsed in two changes of 95% alcohol. Finally, slides were mounted with synthetic resin after quick dehydration in the absolute alcohol. After standard operating, elastic fibers were stained blue to black. The elastic fiber losing area was quantified and expressed as a percentage of EVG negative area (yellow arrow) divided by EVG positive area (blue- black wavy lines) (Figure 1). Quantification for the area percentage of IHC stain was performed by ImageJ version 1.48v image analysis software (National Institutes of Health, MD, USA). Fifty-one human samples were used for the experiments: controls (*n* = 19); mild-to-moderate CKD (*n* = 15); severe CKD (*n* = 17). For image analyses, three random high-resolution fields were obtained in each sample.

### Grade Point of Aortic Arch Calcification (AAC)

The standard chest radiograph was taken in posterior-anterior view. A clinician specializing in thoracic radiology and blinded to patient's clinical data independently reviewed one pre-selected standard chest radiograph obtained from each patient within or as close to the amputation surgery as possible. To determine the severity of AAC detectable on chest radiograph, we used a simple AAC grading system: grade point 1 (no visible calcification), grade point 2 (small spots of calcification or single thin calcification of the aortic knob), grade point 3 (one or more areas of thick calcification, but ≤ 50% of the circular area of the aortic knob), and grade point 4 (circular calcification with > 50% of circular area of the aortic knob).

### Statistics

All data were expressed as the mean ± SD using the GraphPad Prism 7 (GraphPad Software, Inc., CA, USA) or SPSS version 22.0 (IBM, NY, USA). Statistical significance was determined by ANOVA (with Dunnett's multiple comparisons test) for three groups. *p* < 0.05 were considered to be statistically significant.

## Results

The final study sample included 51 patients with amputation specimens and complete medical records. Comparisons of baseline bio-clinical characteristics among three study groups of normal controls, mild-to-moderate and severe CKD were summarized in Table 1. The background bio-demographic characteristics were generally similar among three study groups, except the renal function related profiles. After a careful review of medical records, hepatobiliary diseases were excluded in our patient groups. Thus, raised circulating ALP levels in our study were determined to be non-hepatic origins, such as bone events or CKD-mineral bone diseases (18). Non-hepatic ALP is a byproduct of osteoblast activity, and increased hydrolysis of mineralization inhibitor pyrophosphate by ALP enhances UVC in CKD (19). Our data demonstrated elevated plasma concentrations of non-hepatic ALP, calcium-phosphate product, and CRP were in parallel with eGFR decline among patients with amputations. Elevated concentrations of circulating calcium-phosphate product reflect diffuse precipitation of calcium phosphate in tissues, leading to widespread UVC and organ dysfunction. Above results demonstrated higher levels of pro-calcific stress in uremic milieu upregulated systemic inflammatory responses.

**Table 1:** Comparisons of baseline bio-clinical characteristics among three study groups of normal controls, mild-to-moderate and severe CKD^a^.

**Variables**	**Controls (*n* = 19)**	**Mild-to-moderate CKD (*n* = 15)**	**Severe CKD (*n* = 17)**	***P*-value**
Age (years)	72.2 ± 14.3	70.8 ± 9.7	61.6 ± 13.8	0.04
Male, *n* (%)	14 (73.9)	11 (73.3)	13 (76.5)	0.98
Smoking, *n* (%)	9 (47.4)	7 (46.7)	9 (52.9)	0.93
*AAC grade score*^f^	*1.8 ± 0.7*	*1.9 ± 0.8*	*2.5 ± 0.9*	*0.03*
Systolic blood pressure (mmHg)	131.5 ± 26.6	134.7 ± 25.0	135.0 ± 26.8	0.91
*Diastolic blood pressure (mmHg)*	*73.3 ± 13.5*	*78.9 ± 15.1*	*75.8 ± 11.1*	*0.48*
*Blood urea nitrogen (mg/dL)*	*21.0 ± 9.1*	*48.9 ± 24.2*	*54.9 ± 26.6*	*<0.01*
*Creatinine (mg/dL)*	*0.8 ± 0.2*	*2.0 ± 0.5*	*5.8 ± 2.1*	*<0.01*
*eGFR (mL/min)*^b^	*96.5 ± 27.6*	*35.9 ± 13.3*	*11.4 ± 4.0*	*<0.01*
*Sodium (mmol/L)*	*135.1 ± 5.1*	*135.6 ± 5.9*	*132.7 ± 5.0*	*0.26*
Potassium (mmol/L)	3.9 ± 0.6	4.3 ± 1.2	4.4 ± 0.7	0.27
*Glucose (mg/dL)*	*187.3 ± 121.7*	*305.1 ± 149.7*	*283.7 ± 213.2*	*0.13*
*Alanine aminotransferase (IU/L)*	*27.5 ± 17.5*	*25.7 ± 19.3*	*23.2 ± 19.3*	*0.99*
*Aspartate aminotransferase (IU/L)*	*35.3 ± 25.4*	*33.3 ± 19.0*	*34.5 ± 32.0*	*0.78*
Albumin (g/dL)	2.9 ± 0.6	2.7 ± 0.4	2.8 ± 0.7	0.79
*Non-hepatic alkaline phosphatase (IU/L)*	*136.0 ± 43.2*	*152.7 ± 9.5*	*208.9 ± 78.2*	*0.05*^c^
Calcium (mg/dL)	8.7 ± 0.6	9.3 ± 0.6	9.6 ± 0.9	0.18
Adjusted calcium (mg/dL)^d^	9.3 ± 0.8	10.3 ± 0.9	10.8 ± 1.2	0.12
*Phosphate (mg/dL)*	*3.0 ± 0.4*	*3.8 ± 0.7*	*5.5 ± 2.1*	*0.06*
Calcium-phosphate product^e^	28.0 ± 1.7	39.0 ± 6.5	58.6 ± 19.0	0.02
Total cholesterol (mg/dL)	189.5 ± 48.2	191.1 ± 51.8	182.5 ± 34.1	0.55
Triglyceride (mg/dL)	205.2 ± 179.9	214.1 ± 193.7	177.0 ± 124.2	0.60
*Low density lipoprotein*	*112.5 ± 42.7*	*99.8 ± 7.5*	*74.3 ± 35.2*	*0.30*
C-reactive protein (mg/L)	8.8 ± 7.9	16.8 ± 7.4	22.7 ± 8.1	0.02

*Continuous variables were expressed as mean ± SD*.

*Categorical variables are expressed as n (%)*.

*Non-normally distributed parameters of the whole study population (n = 52) were shown in italics*.

a *CKD, chronic kidney disease. Control groups, eGFR ≧ 60 ml/min; Mild-to-moderate CKD, 15 ml/min < eGFR ≦ 60 ml/min; Severe CKD, eGFR ≦ 15 ml/min*.

b *eGFR, estimated glomerular filtration rate*.

c *p, 0.047*.

d *Adjusted calcium, measured calcium+ (4.0—albumin) × 0.8*.

e *Calcium-phosphate product, adjusted calcium × phosphate*.

f *AAC, Aortic arch calcification*.

### CKD Arteries Exhibited eGFR-Dependent Internal Elastic Lamina (EL) Disruption, Corresponding With UVC Severity

It is unclear how circulating procalcific particles dramatically leak into the arterial medial layer tunica media (TM). Tunica intima (TI), the innermost layer of vessels, is supported by an internal EL. TM is separated from TI by the internal EL, and thereby internal EL is the final line of defense against circulating procalcific particles. Internal EL provided important implications for cellular communications and material transports between the TI and TM. We aimed to provide the evidence that the internal EL in CKD arteries were profoundly eroded in uremic milieu, leading to a leaky intima. In CKD arteries, internal EL disruption was propagated along the media of the arterial wall (Figure 1A). As expected, the area loss from internal EL disruption was correlated with CKD severity. Arteries from basal group appeared histologically intact, whereas CKD arteries exhibited progressive calcium deposition in parallel with eGFR decline (Figure 1B). Taken together, eGFR-dependent UVC corresponded with the internal EL defect area (Figure 1). Stretching, fragmentation and loss of internal ELs were often presented adjacent to UVC regions. Influx of circulating mineral ions into TM layer with subsequent deposition of nanoparticles may develop through breaks in the internal EL. Above results unveiled CKD arteries exhibited eGFR-dependent internal EL disruption, allowing for catastrophic mineral ion influx into TM to promote systemic UVC.

### eGFR-Dependent UVC Is Concordant With Impaired Contractility and Apoptosis of VSMC in TM

**Figure 2:**
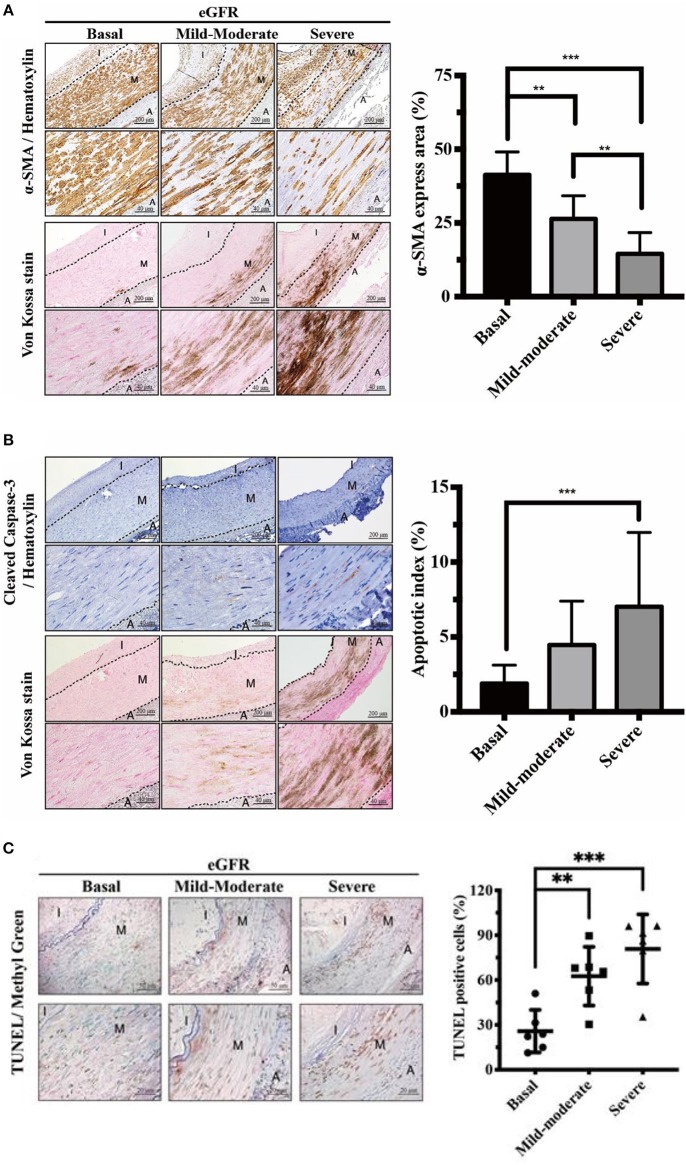
Effects of eGFR-dependent UVC on VSMC contractility impairment and apoptosis in tunica media. Quantitative analysis of immunohistochemical staining for **(A)** α-SMA **(B)** Caspase-3 and **(C)** TUNEL was performed using ImageJ software. Note that the decrease of contractile VSMCs and VSMC apoptosis were presented adjacent to the eGFR-dependent UVC regions. Fifty-one human samples were used for the experiments: controls (*n* = 19); mild-to-moderate CKD (*n* = 15); severe CKD (*n* = 17). For image analyses, three random high-resolution fields were obtained in each sample. Scale bars in the upper panels are 200 and 40 μm in the lower panels. Data are expressed as mean ± SD. **P < 0.01; ***P < 0.001. A, adventitia; eGFR, estimated glomerular filtration rate; I, intima; M, media; SMA, smooth muscle actin; TUNEL, terminal deoxynucleotidyl transferase dUTP nick end labeling; UVC, uremic vascular calcification; VSMC, vascular smooth muscle cells.

With intact internal EL, contractile VSMCs in the normal vascular TM are completely protected from the mineral ion stress and express high levels of contractile proteins such as αSMA. UVC is attributable to VSMC apoptosis, which provides more abundant membrane niduses for calcification, increases local calcium concentrations and further inhibits VSMC's capacity of tissue repair after vascular injury. To outreach above findings to translational research in human muscular arteries, VSMC contractility and viability were evaluated by αSMA stain and caspase-3 assay, respectively. As shown in Figure 2A, eGFR-dependent UVC was inversely correlated the expression of αSMA, indicative of the decrease of contractile VSMCs and arterial stiffness. Figures 2B,C illustrated eGFR-dependent UVC was robustly correlated the expression of caspase-3 and TUNEL, indicative of VSMC apoptosis. Current data demonstrated eGFR-dependent UVC is concordant with VSMC contractility impairment and apoptosis in CKD arteries, leading to further UVC niduses formation and disabling VSMC repair mechanism.

### eGFR-Dependent Runx2 Corresponded With Positive Staining of ALP in VSMC, Indicative of Osteogenic Differentiation

**Figure 3:**
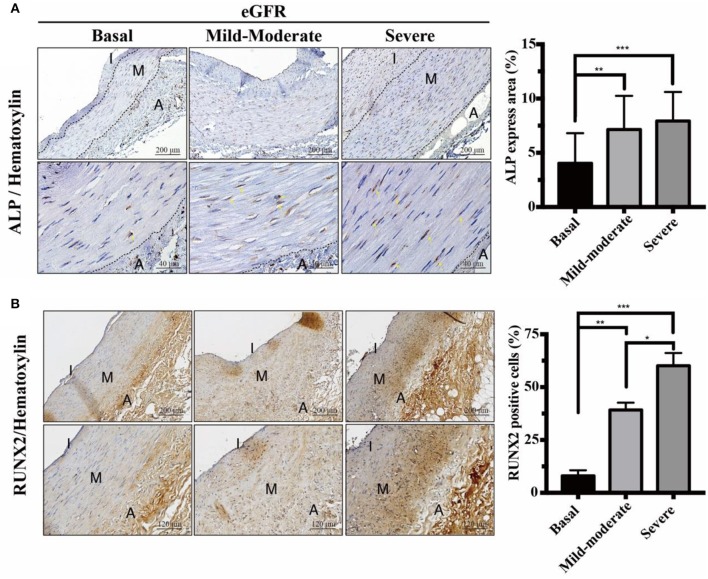
Effects of eGFR-dependent osteogenic transcription factor Runx2 on ALP expressions. Quantitative analysis of immunohistochemical staining for **(A)** ALP and **(B)** Runx2 was performed using ImageJ software. Yellow arrows indicate the positive staining of ALP in VSMCs. Fifty-one human samples were used for the experiments: controls (*n* = 19); mild-to-moderate CKD (*n* = 15); severe CKD (*n* = 17). For image analyses, three random high-resolution fields were obtained in each sample. Scale bars in the upper panels are 200 and **(A)** 40 μm and **(B)** 120 μm in the lower panels. Note that the positive staining areas of ALP and Runx2 were compatible. Data are expressed as mean ± SD. *P < 0.05; **P < 0.01; ***P < 0.001. A, adventitia; ALP, alkaline phosphatase; eGFR, estimated glomerular filtration rate; I, intima; M, media; Runx2, runt-related transcription factor 2.

In response to chronic stimuli of uremic milieu and mineral dysregulation, UVC is reminiscent of osteogenic differentiation in VSMCs (12). Runx2 is upregulated early in the phenotypic transition of VSMCs and acts as an essential transcription factor driving the process. Maladaptive change of VSMCs could initially prolong survival, yet ultimately resulting in apoptosis and progressive UVC and CV events. Further, raised serum non-hepatic ALP levels have been shown to be associated with increased mortality in patients on maintenance hemodialysis (20). To provide pathobiological evidence in support of above findings, our data indicated eGFR-dependent UVC corresponded with higher expressions of ALP and Runx2 (Figure 3). ALP increases in response to active osteocytic conversion of VSMC, as ALP is a byproduct of osteoblast activity. Runx2 acts as a key regulator of osteoblast differentiation and triggers the expression of downstream ALP. Current data demonstrate that active VSMC osteogenic differentiation plays a pivotal role in the pathobiology of progressive UVC, and it is different from passive mineral deposition in TM due to the leaky intima.

### In Accordance With UVC Severity, CKD Arteries Exhibited eGFR-Dependent Increases in 8-OHdG Expressions, Indicative of Oxidative Injury

**Figure 4:**
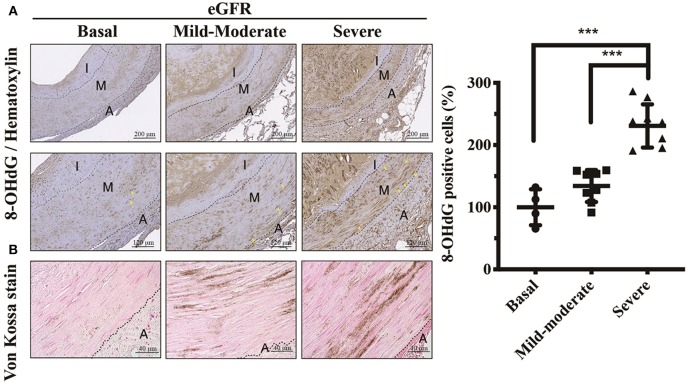
Effects of eGFR-dependent UVC on oxidative DNA damages of VSMCs. **(A)** Vascular oxidative injury was examined by immunohistochemical staining of 8-OHdG. Yellow arrows indicate positive staining of 8-OHdG in nuclei of VSMCs. **(B)** Calcium deposits were localized using Von Kossa stain. Scale bars in the upper panels are 200 μm, and **(A)** 120 μm and **(B)** 40 μm in the lower panels. Fifty-one human samples were used for the experiments: controls (*n* = 19); mild-to-moderate CKD (*n* = 15); severe CKD (*n* = 17). For image analyses, three random high-resolution fields were obtained in each sample. Quantitative analysis for positive staining was performed using ImageJ software. Data are expressed as mean ± SD. ***P < 0.001. 8-OHdG, 8-hydroxy-2'-deoxyguanosine; A, adventitia; eGFR, estimated glomerular filtration rate; I, intima; M, media; UVC, uremic vascular calcification; VSMC, vascular smooth muscle cell.

The uremic milieu consists of plenteous toxins that exert pro-oxidant effects. Uremic toxins have been reported to cause alveolo-capillary injury through triggering ROS to activate downstream inflammatory signaling pathways, and antioxidant therapy could ameliorate organ damages (21, 22). To investigate it further, we outreached above findings to current translational cardiovascular research. As expected, CKD arteries exhibited eGFR-dependent increases in 8-OHdG expressions, indicative of DNA damages of VSMCs due to ROS (Figure 4). The regions involved in oxidative DNA damages of VSMCs were concordant with UVC areas. After direct exposure to uremic toxins, ROS initiates tissue damages by inducing DNA modifications that act to inhibit normal VSMC repair functions, and further undergo maladaptive phenotypic transition (16, 23). Taken together, the results of the current study illustrate ROS serves as key messenger molecules, resulting in vascular oxidative injury, VSMC osteogenic differentiation, apoptosis and irremediable UVC.

### UVC Is Correlated With eGFR, Internal EL Disruption, Osteogenic Differentiation, and Oxidative Damage

**Figure 5:**
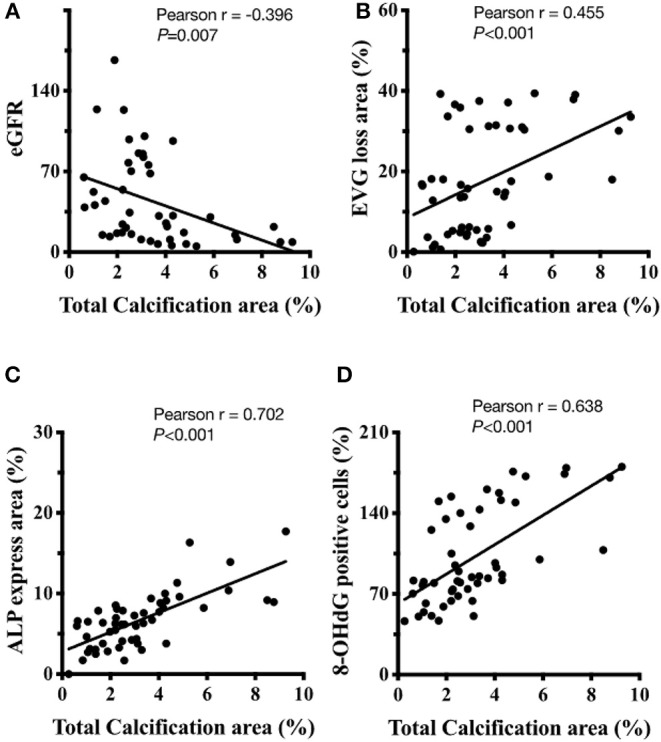
The correlation analysis between UVC, eGFR, internal EL disruption, osteogenic differentiation, and oxidative damage. **(A)** The correlation coefficient *r* between vascular calcification area and eGFR is−0.396 (*P* < 0.01). **(B)** The correlation coefficient *r* between vascular calcification area and EVG loss area is 0.455 (*P* < 0.01). **(C)** The correlation coefficient *r* between vascular calcification area and ALP expression is 0.702 (*P* < 0.01). **(D)** The correlation coefficient *r* between vascular calcification area and 8-OHdG positive cells is 0.638 (*P* < 0.01). Fifty-one human samples were used for the experiments. Quantitative analysis for positive staining was performed using ImageJ software. 8-OHdG, 8-hydroxy-2′-deoxyguanosine; eGFR, ALP, alkaline phosphatase; estimated glomerular filtration rate; EVG, elastic tissue fibers—Verhoeff's Van Gieson stain.

We conducted a correlation analysis between UVC, eGFR, internal EL disruption, osteogenic differentiation and oxidative damage. The scatter diagrams (Figure 5) indicated correlations between vascular calcification area, eGFR decline, EVG loss area, ALP expressions and 8-OHdG positive cells were robust. The correlation coefficient *r* between vascular calcification area and eGFR is −0.396 (*P* < 0.01). The correlation coefficient *r* between vascular calcification area and EVG loss area is 0.455 (*P* < 0.01). The correlation coefficient *r* between vascular calcification area and ALP expression is 0.702 (*P* < 0.01). The correlation coefficient *r* between vascular calcification area and 8-OHdG positive cells is 0.638 (*P* < 0.01). Above results provided the evidence that the severity of UVC is eGFR-dependent and significantly correlated with internal EL disruption, VSMC re-programming and oxidative damage.

Our study has several limitations. Firstly, our study sample size was relatively small. Next, cross-sectional laboratory values might not reflect substantial intra-individual variability over time. Last but not least, our study using pathology slides for retrospective analysis is subject to residual confounders. To link uremia and vascular calcification, serum from CKD patients should be used to treat human VSMCs and arterial rings *ex vivo*, and determine whether this would trigger vascular oxidative stress, leading to progressive internal EL disruption, calcification and apoptosis.

## Discussion

UVC process, the deposition of hydroxyapatite mineral in the arterial TM layer, had been viewed as binary: active reprogramming of VSMC and passive mineral deposition in ECM. Why are CKD patients prone to suffer from UVC? Evidence is emerging in recent years that UVC is principally driven by VSMC in response to active inducers of mineralization, including hypercalcemia, hyperphosphatemia, uremic toxins, oxidative stress, and multiplex inflammatory mediators. Even though the paracellular ion transport is passive diffusion and dependent on the concentration gradient, underneath of intima is internal EL affecting molecular transport across the TM layer (24). Since internal EL is a dense elastic fiber layer as the defense line in protecting of the integrity of arterial wall, it is difficult for circulating mineralization inducers to leak into TM layer and drive active reprogramming of VSMC. To gain pathobiological insights into the initiation of UVC process, we used human models of medium-sized muscular arteries with tissue staining procedures. Several important issues deserve further discussion in this cardiorenal translational research.

### Oxidative Injury Induced Internal EL Disruption as an Emerging Key Player of UVC Progression

A myriad of toxins promote oxidative stress in the uremic milieu (15, 17). Such oxidative stress initiates tissue damages by inducing modifications in both proteins and DNA (16), contributing to the abnormal structures and functions of elastic fibers in pathological conditions (25). To the best of our knowledge, this is the first study to demonstrate oxidative DNA damage (8-OhdG) of VSMC corresponds with the deterioration of renal function and disruption of internal EL. The eGFR-dependent internal EL disruption acts as growing breakpoints to allow an influx of circulating mineral ions into TM to induce VSMC osteogenesis, apoptosis, and overwhelming UVC. Our data unravel the mystery of UVC progression that vascular oxidative injury triggered by uremic toxins results in internal EL disruption as a key event in active VSMC reprogramming and passive mineral deposition in ECM. In light of this, oxidative EL injury might be the rate-determining step that induces catastrophic mineral ion influx into VSMC layer, ultimately irremediable UVC.

### Oxidative Stress Drives VSMC Apoptosis, αSMA Loss and Osteogenic Differentiation

The cellular source of ROS is mainly from the mitochondria during oxidative phosphorylation, and aberrant calcium influx results in mitochondrial stress (26). Higher mitochondrial metabolism and energy consumption provided by mitochondrial respiration are also required for osteogenic differentiation of VSMC (26). In addition to energy production, mitochondria serve as a pivotal role in mediating cell apoptosis through caspase-3 activation (27). Mitochondrial dysfunction, oxidative stress, and apoptosis are well-known drivers of UVC (23), and ameliorating mitochondrial-related oxidative stress inhibits cell apoptosis (28). As expected, our data indicate upregulated expressions of cleaved caspase-3 and TUNEL are associated with CKD progression and αSMA loss (11). Furthermore, VSMC apoptosis provides more abundant membrane niduses for UVC, which is unfavorable to tissue repair after vascular injury. In normal vessels, VSMC are capable of contracting and express high levels of contractile markers such as αSMA. After exposure to oxidative injury, VSMC become synthetic and proliferative to repair vessels, accompanied by down-regulation of contractile proteins (10). Clinical complications of contractile protein loss include arterial stiffness, widened pulse pressure, increased pulse wave velocity, and raised all-cause mortality (23). Under chronic stimuli of mineral dysregulation and uremic toxins, synthetic VSMC undergo further maladaptive osteocytic conversion and express markers normally restricted to bone, such as the osteogenic transcription factors Runx2 and the mineralization regulating proteins ALP. ALP is ubiquitously upregulated in the earliest stages of VSMC calcification and degrades the calcification inhibitor pyrophosphate. It has been well-documented ROS accumulation modulates the expression of osteogenic transcription factor Runx2 (29), which in turn induces the osteocytic conversion of VSMC. Collectively, our results demonstrate CKD arteries exhibit eGFR-dependent vascular oxidative injury, corresponding with expressions of VSMC apoptosis, αSMA loss and osteogenic differentiation.

### Pro-Calcific Phenotype as a Temporary Survival Mechanism of VSMC

In normal physiology, DNA damages evoke intricate signaling pathways to repair DNA strand breaks that constantly occur in response to environmental stimuli (30). Nonetheless, if DNA damages become persistent and irreparable due to overwhelming oxidative injury, VSMCs undergo senescence and secrete cytokines and growth factors that promote osteogenic differentiation (31). Of note, just prior to senescence, VSMC enhance ALP activity and Runx2 expression, which suggests that cellular aging may induce a particular “pro-calcific phenotype” and drive calcification within the vascular wall (32). In support of this notion, our data demonstrate eGFR-dependent DNA damage (8-OHdG) of VSMC is in parallel with ALP activity and Runx2 expression. These adaptations may temporarily extend the survival of VSMC but ultimately culminate in cell death and irremediable UVC.

## Conclusion

**Figure 6:**
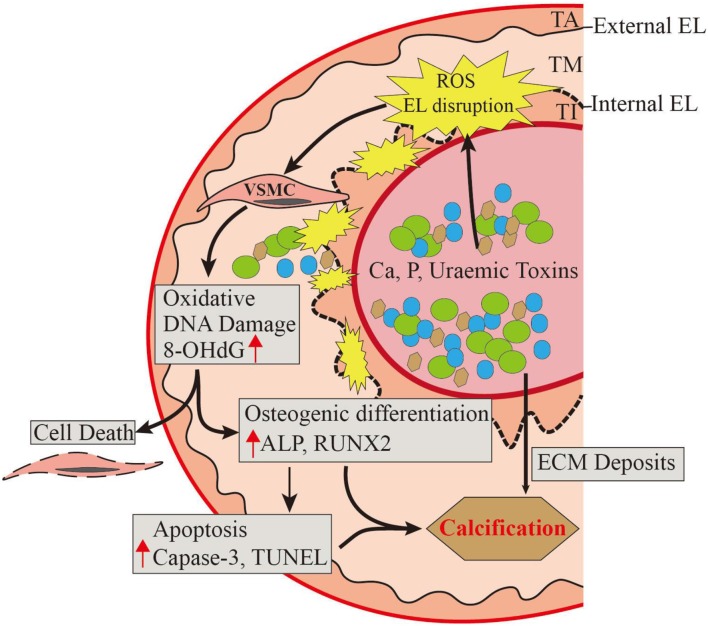
A comprehensive pathobiological insight into CKD-driven UVC. The direct exposure to circulating uremic milieu triggers vascular oxidative injury, leading to EL disruption as the key event in disabling VSMC defense mechanisms. Loss of intima integrity might be the rate-determining step in early stage of CKD, corresponding with oxidative VSMC re-programming, apoptosis, and ultimately irremediable UVC. Oxidative EL injury induces catastrophic mineral ion influx into VSMC layer, thus novel antioxidants will be of vital importance in treating UVC related cardiovascular events. ALP, alkaline phosphatase; Ca, calcium cations; CKD, chronic kidney disease; ECM, extracellular matrix; EL, elastic lamina; P, phosphate anions; TA, tunica adventitia; TI, tunica intima; TM, tunica media; ROS, reactive oxygen species; Runx2, runt-related transcription factor 2; UVC, uremic vascular calcification; VSMC, vascular smooth muscle cell. 8-OHdG, 8-hydroxy-2′-deoxyguanosine.

UVC, a common and inevitable complication in CKD patients, is predictive of CV events. Our research in human vascular tissues contributes comprehensive pathobiological insights into CKD-driven UVC (Figure 6). The direct exposure to circulating uremic milieu triggers vascular oxidative stress, leading to EL disruption as a key event in disabling VSMC defense mechanisms. EL disruption and oxidative DNA damage begin in early stage of CKD, corresponding with active VSMC re-programming and apoptosis. Loss of intima integrity might be the rate-determining step that allows more nanocrystals to leak into medial layer, ultimately irremediable UVC. In light of this, novel antioxidant therapies will be of vital importance in preventing UVC related cardiovascular events.

## Data Availability Statement

The datasets for this article are not publicly available because they contain information that could compromise the privacy of research participants. Requests to access the datasets should be directed to Jia-Feng Chang, cjf6699@gmail.com.

## Ethics Statement

The studies involving human participants were reviewed and approved by the Research Ethics Review Committee of the En Chu Kong Hospital (ECKH1050402). Written informed consent for participation was not required for this study in accordance with the national legislation and the institutional requirements.

## Author Contributions

J-FC and S-HL were responsible for study concept and design, interpretation of data, writing of the manuscript, study supervision, and drafting of the manuscript. C-TH and C-YH assisted with biochemical and digital analysis. S-MK, C-CW, L-LW, J-CL, and S-WC provided experimental supports. T-MW, W-NL, and K-CL were responsible for the manuscript revision. S-HL assisted with the morphometric analysis in histopathology. Y-YL conceived the original idea and suggested the experimental work.

### Conflict of Interest

The authors declare that the research was conducted in the absence of any commercial or financial relationships that could be construed as a potential conflict of interest.
